# Assessment of Treatment Effects of Aminaphtone by Capillaroscopy in a Patient with Raynaud’s Phenomenon

**DOI:** 10.3390/ph18020203

**Published:** 2025-02-02

**Authors:** Gianluca Screm, Lucrezia Mondini, Francesco Salton, Paola Confalonieri, Chiara Bozzi, Chiara Torregiani, Caterina Antonaglia, Pietro Geri, Mario D’Oria, Giulia Bandini, Michael Hughes, Marco Confalonieri, Barbara Ruaro

**Affiliations:** 1 Pulmonology Unit, Department of Medical Surgical and Health Sciences, Hospital of Cattinara, University of Trieste, 34149 Trieste, Italy; 2 Division of Vascular and Endovascular Surgery, Department of Clinical Surgical and Health Sciences, University of Trieste, 34149 Trieste, Italy; 3 Department of Experimental and Clinical Medicine, Division of Internal Medicine, Azienda Ospedaliero Universitaria Careggi, University of Florence, 50134 Florence, Italy; 4 Division of Musculoskeletal and Dermatological Sciences, Faculty of Biology, Medicine and Health, Salford Royal NHS Foundation Trust, The University of Manchester, Manchester M6 8HD, UK

**Keywords:** aminaphtone, nailfold video capillaroscopy (NVC), capillary disorders

## Abstract

**Background:** Aminaphtone is a well-established pharmaceutical agent that has been utilized for over 40 years, primarily due to its effectiveness in treating microvascular disorders. Recent studies have explored its impact on various conditions, including chronic venous insufficiency, diabetic microangiopathy, leg ulcers, systemic sclerosis, and Raynaud’s phenomenon. These investigations have consistently demonstrated that aminaphtone enhances skin blood perfusion and mitigates endothelial damage, all while maintaining a robust safety profile over time. **Case Summary:** This report highlights the potential of aminaphtone in improving microcirculation in a young patient who experienced spontaneous capillary rupture in her second finger. A 38-year-old woman with undifferentiated connective tissue disease presented to the clinic for periungual videocapillaroscopy (NVC). Given the microangiopathic changes observed during the NVC, she was prescribed aminaphtone. After seven months of treatment, a follow-up NVC revealed significant improvement in the capillaroscopic findings. A comprehensive literature review on aminaphtone was conducted using electronic databases (PUBMED, Google Scholar, ResearchGate, UpToDate), along with manual searches, focusing on articles published until November 2024. **Conclusion:** Treatment with aminaphtone led to notable improvements in microangiopathic health. Following the introduction of this medication, the nailfold microvascular bed, which previously exhibited severe alterations, showed a remarkable transition to only mild abnormalities.

## 1. Introduction

Nailfold video capillaroscopy (NVC) is a diagnostic technique designed to establish a standardized approach for diagnosing and monitoring various diseases by evaluating the small vessels of the microcirculation [1,2,3]. Currently, it plays a significant role in assessing several autoimmune conditions, such as systemic sclerosis and Raynaud’s phenomenon (RP) [1,2,4]. However, its potential applications are also being explored in other areas.

**Figure 1:**
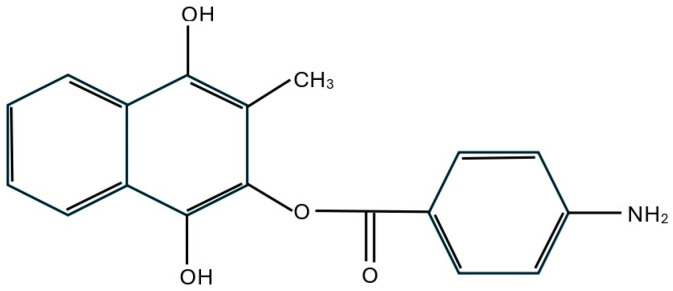
Structure formula of aminaphtone (C18-H15-N-O4).

Aminaphtone is a chemical compound derived from 4-aminobenzoic acid (2-hydroxy-3-methyl-1,4-naphthohydroquinone-2-p-aminobenzoate) and is primarily used for treating capillary disorders [5,6,7]. The structural formula of aminaphtone is presented in Figure 1 [6,7,8,9,10].

It has a mechanism of action that is based on interference with several biological molecules that are involved in the control of endothelial hemostasis [11,12,13]. Its main role is to reduce endothelin-1 production, and this drug seems to have an important clinical impact regarding the improvement of the symptoms associated with RP [6,13,14].

## 2. Case Presentation

The patient’s clinical history began in 2018 at a hospital in another city, where they experienced dry eyes following a common viral infection and a Schirmer’s test was performed, which returned positive results. Subsequently, the patient received a rheumatological evaluation. During the physical examination, the doctor checked for swelling in the parotid or other salivary glands, joint swelling, and any skin or neurological symptoms that might suggest a rheumatic disorder like Sjogren’s syndrome. However, the examination results were normal. Laboratory tests indicated negative results for antinuclear antibodies and other antibodies, leading to the recommendation of lubricating eye drops (Systane Ultra) without any further intervention at that time. In 2022, due to persistent symptoms, an ultrasound of the parotid and major salivary glands was ordered in another research center, but the results were inconclusive for Sjogren’s syndrome. Additional lab tests, prescribed to complete the assessment of patient, showed only positive antinuclear antibodies (titer 1:320, with a dense fine speckled indirect immunofluorescence pattern) and mild neutropenia. The other tests for renal and liver function, inflammatory markers (like erythrocyte sedimentation rate, C-reactive protein, and complement fraction), rheumatoid factor, and various autoimmune markers (including ENA, anti-ds-DNA, antiphospholipid antibodies, and lupus anticoagulant) were normal. A minor salivary gland biopsy was conducted, which did not reveal significant lesions. Overall, the patient was diagnosed with undifferentiated connective tissue disease. In March 2024, when she was 38 years old, she experienced a spontaneous rupture of the capillaries of the second digit of the right hand. Due to this event, a periungual videocapillaroscopy (NVC) was requested and performed in our center.

**Figure 2:**
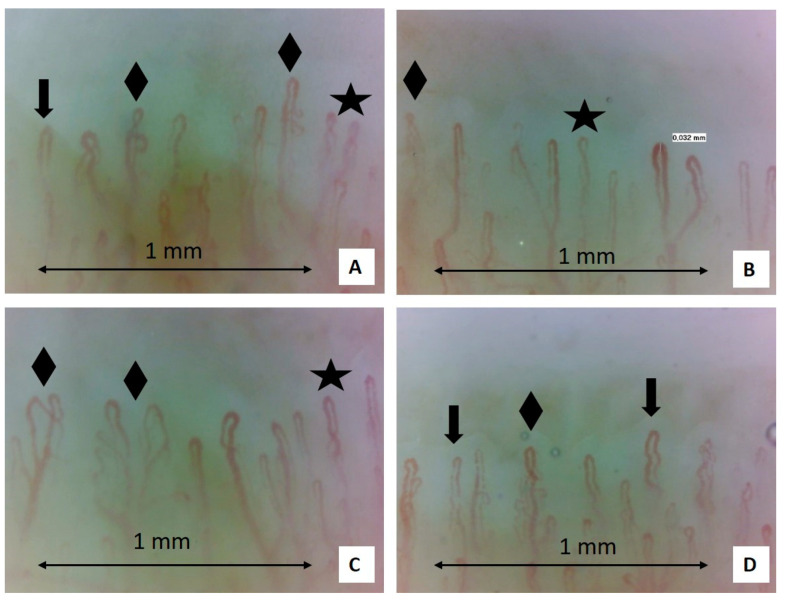
Nailfold video capillaroscopy images recorded before treatment with aminaphtone (**A**–**D**) (magnification: 200×). Black stars (**A**–**C**) indicate the presence of crossing capillaries. Black arrows (**A**,**D**) indicate the tortuosity of capillaries. Black rhombuses (**A**–**D**) show the presence of ramified capillaries. In these figures, the capillary count per linear mm results in 7 capillaries in 1 mm (slightly reduced) [16,17] (Operators: B.R. and L. M., Pulmonology Unit, University of Trieste).

The NVC demonstrated several microangiopathic alterations. There were many crossed and tortuous capillaries present, as well as many enlarged capillaries [15,16]. Capillary hemorrhages were found on the second digit of the right hand, which were not linked to traumatic causes due to their morphology. A relative reduction in capillary flow and a slight presence of edema were also found (Figure 2). Furthermore, Raynaud’s condition score (RCS, which is a comprehensive assessment given by the patient regarding the frequency and duration of attacks, pain, and functional impairment related to RP) was assessed using a visual analogue scale (VAS: none (0)–very severe (100)]), and the value was 80/100 [4].

Due to this clinical evidence, aminaphtone therapy was started. The patient took one tablet of aminaphtone per day for 7 months: to the best of our knowledge, the patient declared adherence to the treatment and no side effects of the drug.

**Figure 3:**
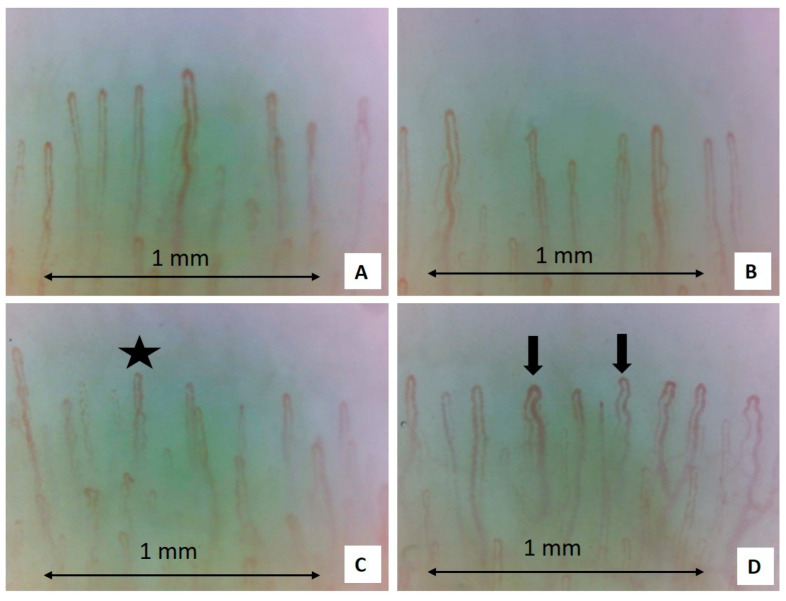
Nailfold video capillaroscopy images recorded after treatment with capillarema (magnification: 200×). Black stars (**C**) indicate the presence of crossing capillaries. Black arrows (**D**) indicate the tortuosity of capillaries. In these figures, the capillary count per linear mm results in 8 capillaries in 1 mm (normal number) (**A**–**D**) [16,17] (Operators: B.R. and L.M., Pulmonology Unit, University of Trieste).

After treatment with aminaphtone, nailfold video capillaroscopy was performed again, and the overall capillaroscopic picture was strongly improved. Crossed, tortuous, and enlarged capillaries were detected rarely. Capillary hemorrhages had disappeared, and blood flow was normal. Only the slight presence of edema persisted (Figure 3). Furthermore, the RCS value was 40/100.

## 3. Discussion

This is the first case report that has evaluated the effects of aminaphtone treatment on both capillaroscopy alterations and clinical symptoms in patients affected by secondary Raynaud’s phenomenon (RP). In the literature, several articles have reported that the effects of aminaphtone treatment lead to progressive improvements concerning microvascular dysfunction in several diseases [7,8,9,10,11,12]. This drug works through interferences against molecules which have multiple roles in microcirculation, so it acts through the downregulation of several adhesion molecules, pro-inflammatory cytokines, and vasoconstriction molecules (especially endothelin-1 production) [5,10,13]. When the endothelium goes through any kind of trauma, the outcome is an over-expression of several molecules, most of those being the ones targeted by the drug. The result is a vasoconstriction and pro-inflammatory response, along with the promotion of migration and adhesion of several molecules from the bloodstream [14]. Consequently, aminaphtone has been shown to be more effective in those disease, which are listened in Table 1, where microvascular complications are often due to dysregulation of these molecules.

Looking at the impactful articles listed above, it can be easily argued that the efficacy of aminaphtone treatment is heavily tangible, especially in the management of RP symptoms and in the improvement of patients’ quality of life. It has already been established that the application field of aminaphtone is restricted to certain diseases. Nevertheless, as this case reports, whatever the patient’s medical condition, the manifestations of any minor peripheral microangiopathic complications may be monitored after taking this drug [5,6,7].

Raynaud’s phenomenon has a profound effect on the quality of life of those affected. Not only does it cause significant pain, but it also creates significant difficulty in carrying out daily activities [8,9,10]. While existing treatments are generally considered tolerable, they rarely provide complete relief, highlighting a critical need for new treatment options. Management strategies for this condition are primarily informed by existing literature, expert opinion, and current clinical practice [11,12,13]. Given the cost and practicality considerations, EULAR experts recommended that calcium antagonists serve as a first-line treatment for secondary Raynaud’s phenomenon associated with systemic sclerosis, while intravenous prostanoids should be considered if calcium antagonists are ineffective [14,17,18]. Because of the potential vascular side effects associated with both types of drugs, careful monitoring is advised, especially when combining prostanoids with calcium channel blockers. Recently, increased availability and interest in nailfold capillaroscopy conducted by assessing morphological capillary/microcirculatory variations has paved the way for studies on early intervention and vascular protection in RP patients [7,11,13].

Our case report emphasizes the efficacy of aminaphtone in treating Raynaud’s phenomenon (RP). Although the exact mechanisms of action of aminaphtone remain unclear, recent studies indicate that it reduces vascular permeability and tissue edema [7,11,12].

Additionally, in vitro research suggests that aminaphtone may down-regulate the expression of E-selectin (ELAM-1), Vascular Cell Adhesion Molecule-1 (VCAM-1), and Intercellular Adhesion Molecule-1 (ICAM-1), as well as modulate the production of cytokines, chemokines, and endothelin-1 in cultured human endothelial cells [6,10]. Aminaphtone appears to reduce the transcription and protein production of key inflammatory mediators, including IL-6 [6,10], and it also lowers TGF-β levels, which can contribute to pulmonary fibrosis by stimulating fibroblasts to excessively deposit collagen [5,6,7]. These actions may support the findings of several studies that have reported the potential benefits of aminaphtone in conjunction with standard therapy for RP patients, suggesting a synergistic effect on vasospastic episodes [7,11,12]. According to the technical data sheet, aminaphtone is partially metabolized to phthiocol in humans and is eliminated via the urine within 72 h, with peak excretion occurring six hours post-administration. Preclinical safety evaluations, including acute toxicity studies across four animal species at doses up to 3 g/kg, subacute toxicity in two species at doses up to 100 mg/kg for 90 days, and chronic toxicity in dogs at 50 mg/kg for 280 days, revealed no significant tissue damage or alterations in organ function. Furthermore, aminaphtone has shown no teratogenic or mutagenic effects [6,7,8,9].

The main limitations of our article, common to all case reports, are the evaluation of a single case, the lack of a control case, and the retrospective design. Obviously, further studies are needed in order to better understand the opportunities to establish more effective therapy, along with more rapid resolution of symptoms, with the administration of aminaphtone whenever microangiopathic abnormalities are detected.

## 4. Conclusions

The study demonstrates that aminaphtone treatment ameliorates RP’s clinical symptoms and capillaroscopic alterations, with sustained efficacy until 7 months.

In clinical practice, the administration of aminaphtone to all patients who are detected to have microangiopathic complications during NVC examinations should be considered.

The use of aminaphtone whenever nailfold video capillaroscopy shows any significant microvascular alterations could lead to an increase in the chance of successfully preventing microvascular manifestations of several autoimmune diseases.

## Figures and Tables

**Table 1:** Variation in microangiopathy and clinical symptoms after aminaphtone administration.

Disease	Study	Effects of Aminaphtone Treatment
Chronic venous insufficiency	[8]	This study described the efficacy of aminaphtone treatment compared to a control group. The aminaphtone cohort showed significant improvements in terms of quality of life compared to the control group.
Diabetes	[9]	This article reported the case of a patient affected by type I diabetes with an early manifestation of diabetic microangiopathy (define by pathological microalbuminuria values). The introduction of aminaphtone therapy enhanced renal function, with decreased levels of proteinuria, along with improvement in the capillaroscopic images.
Raynaud phenomenon (RP)	[7]	This article strongly demonstrated that aminaphtone therapy can be key to managing the RP exacerbations (reduced intensity and frequency of attacks). In addition, the administration of this drug improved the patient’s quality of life, as it allowed for better control of symptoms.
Systemic sclerosis (SSc)	[6]	In this small study, 12 SSc patients were administered aminaphtone. The cohort study showed a reduction in adhesion molecules compared to the control group. The overexpression of adhesion molecules defined the damage to the endothelium, which is why this drug may help in managing microangiopathic collateral events.
	[11]	This article described the efficacy of aminaphtone in reducing the rates of secondary-RP attacks in SSc patients.
	[12]	In this care report, the administration of aminaphtone among with the standard therapy significantly improved the RP symptoms, as well as the skin blood perfusion.

## Data Availability

Deidentified participant data will be made available upon motivated request to the Corresponding Author.
